# 81 Post-Burn Psychosocial Outcomes in Pediatric Minority Patients: A Burn Database Cross-sectional Study

**DOI:** 10.1093/jbcr/irad045.055

**Published:** 2023-05-15

**Authors:** Paul Won, Li Ding, Kara McMullen, Haig Yenikomshian

**Affiliations:** University of Southern California, Pasadena, California; University of California, Los Angeles, California; University of Washington, Seattle, Washington; University of Southern California, Los Angeles, California; University of Southern California, Pasadena, California; University of California, Los Angeles, California; University of Washington, Seattle, Washington; University of Southern California, Los Angeles, California; University of Southern California, Pasadena, California; University of California, Los Angeles, California; University of Washington, Seattle, Washington; University of Southern California, Los Angeles, California; University of Southern California, Pasadena, California; University of California, Los Angeles, California; University of Washington, Seattle, Washington; University of Southern California, Los Angeles, California

## Abstract

**Introduction:**

Racial and ethnic minority burn patients often face barriers to longitudinal psychosocial support after injury. Studies utilizing the Burn Model System (BMS) National Database show adult minority patients report worse psychosocial outcomes during burn recovery in domains such as body image or social re-integration. No study to date has investigated differences in psychosocial outcomes by racial or ethnic category in the pediatric population using the BMS database. This study examines eight outcomes in minority pediatric patients to better characterize potential differences in the pediatric patient population.

**Methods:**

Patients under 18 years old treated for burn injury between 2009 and 2019 consented to participate in a multi-center longitudinal study were included in the current sample. We analyzed eight outcomes using the National Institute of Health Toolbox and PROMIS-25 measures with higher reported scores indicating worse symptom severity. Multi-level, linear mixed effects, regression modeling analyzed associations between race/ethnicity and outcomes at three time points (discharge after index hospitalization, 6- and 12- month post-injury.

**Results:**

The total cohort of 277 patients were 81% male (n = 182) and on average 8.78 years old (range: 3-18 years at time of injury). When surveyed 12 months after injury, pediatric Hispanic patients reported a mean fatigue score of 45.8 (95% confidence interval (CI): 41.6, 49.9) compared to pediatric non-Hispanic patient score of 43.0 (95% CI: 37.1, 48.9). At 12 months, Hispanic patients reported a mean anxiety score of 46.1 (95% CI: 42.7, 49.5), compared to 41.4 (95% CI: 25.8, 47.1) reported by non-Hispanic patients. At 12 months after injury, Hispanic patients reported a mean peer relationship score of 51.1 (95% CI: 47.2, 55.1) compared to non-Hispanic patients' score of 55.5 (95% CI: 50.3, 60.6). Hispanic patients also reported a mean pain interference score of 44.7 (95% CI: 42.3, 47.0) compared to non-Hispanic patients' score of 38.3 (95% CI: 34.9, 41.6) at 12 months after injury.

**Conclusions:**

Following burn injury, pediatric Hispanic patients report worse outcomes in several psychosocial domains compared to pediatric non-Hispanic patients, although differences were not statistically significant. However, as significant differences in outcomes exist in adult Hispanic/Latino patients, this study supports the need for further evaluation into potential factors that lead to worse outcomes in Hispanic populations.

**Applicability of Research to Practice:**

Although pediatric Hispanic patients do not report significantly worse outcomes than non-Hispanic patients in short term burn recovery, these patients may benefit from further counseling and psychosocial support after injury.

